# The Radiological Anatomy of the Distal Tibiofibular Joint: A Retrospective Computed Tomography Study

**DOI:** 10.7759/cureus.53540

**Published:** 2024-02-04

**Authors:** Meletis Rozis, Evangelos Sakellariou, Elias Vasiliadis, John Vlamis, Spyros G Pneumaticos

**Affiliations:** 1 3rd Orthopedic Department, National and Kapodistrian University of Athens, KAT General Hospital, Athens, GRC; 2 3rd Orthopedic Department, National and Kapodistrian University of Athens, KAT Trauma Hospital, Athens, GRC

**Keywords:** foot and ankle surgery, ankle fractures, incisura fibularis, tibiofibular joint, syndesmosis

## Abstract

Introduction

Distal tibiofibular joint (DTFJ) injuries are commonly encountered in patients with ankle fractures. Achieving optimal fixation is mandatory, but it requires a thorough understanding of the local anatomical relationships. For this reason, we performed a retrospective CT study in healthy ankles to radiologically describe the normal anatomy of the DTFJ and the anatomical relationship of the fibula within the ankle joint.

Materials and methods

For this study, we retrospectively examined 60 CT scans of healthy, non-injured ankles in a plantigrade position. Patients with prior ankle surgery or systemic diseases with ankle involvement were excluded because we needed to describe the normal anatomy of the joint. The radiological evaluation included the position of the fibula in the fibular notch and the rotational relationship of the fibula with the talus and the medial malleolus.

Results

Our study included 60 healthy ankles. Thirty-three were right ankles, and 27 were left. The cohort included 36 females and 24 males with a mean age of 48.3 years old. We found that the fibular notch was retroverted on the transverse plane, with the tibiofibular engagement being 0.11 mm (SD=1.57 mm, SE=0.2 mm), at 1 cm proximally to the tibial plafond. Additionally, we observed that the fibula was internally rotated against the lateral talar facet, while the medial and lateral malleolus facets were externally rotated in between. Moreover, we found a strong positive correlation between the incisura retroversion and fibular engagement at 1 cm above the tibial plafond line (Pearson correlation=0.273, p=0.03).

Conclusion

Our study highlights the importance of gaining a comprehensive understanding of the inherent anatomy of the DTFJ to achieve reduction goals in ankle fractures. According to our results, in ankle fracture treatment, surgeons should aim for anatomical fracture and syndesmotic fixation, with the fibula in internal rotation against the lateral talar facet. Additionally, as normal tibiofibular engagement is borderline, we do not suggest that over-tightening the syndesmotic screws is essential. This study's findings can aid surgeons in reducing the malreduction rates in patients with ankle fractures.

## Introduction

Distal tibiofibular joint (DTFJ) injury is commonly involved in ankle fractures [1], having a direct impact on quality of life and recovery [2]. DTFJ malreduction results in reduced tibiotalar contact area and worse clinical outcomes [3]. Although optimal fixation means have been long debated in the literature [4-6], the importance of anatomical reduction is strongly recommended [7,8].

In the literature, there are several proposed radiological indices with the potential to indicate a proper reduction and fixation of the DTFJ [9,10]. Nevertheless, in a cadaveric study by Pneumaticos et al., those measurements and radiological relationships between the tibia and fibula were found to be directly influenced by the rotation of the ankle on the radiological views, and, thus, were not reproducible [11].

As an intraoperative radiological evaluation of the DTFJ results in high malediction rates [12], anatomical landmarks, including the visualization of the anterior fibular notch as an attempt to centralize the fibula in its groove, have also been proposed and proven to provide high-reduction quality in unstable DTFJ injuries [13]. In our recent publication [14], we identified specific landmarks and anatomical relationships of the anterior DTFJ, describing a novel reduction pattern. Although 6/7 of the radiologically tested parameters of the operated ankles did not significantly differ from the contralateral, healthy ones, our results showed that we still had a slight discrepancy in fibula rotation compared to normal ankles.

Considering the DTFJ malreduction rates and their clinical impact, we supposed that obtaining more information about normal radiological anatomy would further help us achieve better reductions and limit the complication rates. Thus, we performed a computed tomography (CT) study on healthy ankles to better understand the local anatomy and define the goals of optimal DTFJ reduction.

## Materials and methods

For the study period between 2021 and 2022, we retrospectively evaluated CT images from 60 healthy ankles obtained in the Orthopedic Department of our trauma center hospital. Patients with past injuries or operations in the evaluated ankle were excluded. Additionally, we only enrolled patients without any systemic diseases that could potentially involve the examined ankle. The radiological measurements were made according to Boszczyk et al.’s original works [15,16]. The foot was in a plantigrade position in all patients. For the incisura assessment, the evaluation was typically made at 1 cm above the tibial plafond. Rotational examination and the relationship between the fibula, the talus, and the medial malleolus were made at the ankle joint level.

All patients were informed about the study's purpose and provided informed consent. The study was approved by our Hospital's ethical committee.

Measurements of the DTFJ

**Figure 1:**
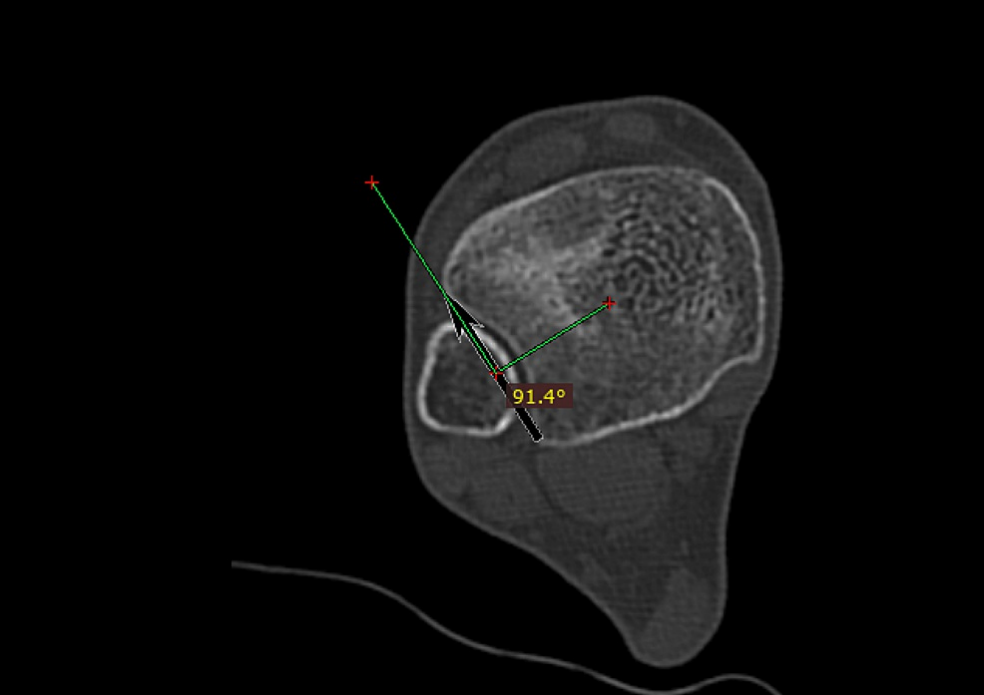
Incisura rotation. We drew a line (baseline) connecting the tibia center with the incisura line (black arrow) center. The angle between the baseline vertical axis and the incisura line is the incisura rotation. In this case, there is a 1.4° of retroversion.

Incisura rotation: Refers to the version of the incisura on the transverse plane. We drew a line from the tibia's center to the incisura line's center (baseline). The incisura rotation was the angle between the baseline vertical axis and incisura line (Figure 1). 

**Figure 2:**
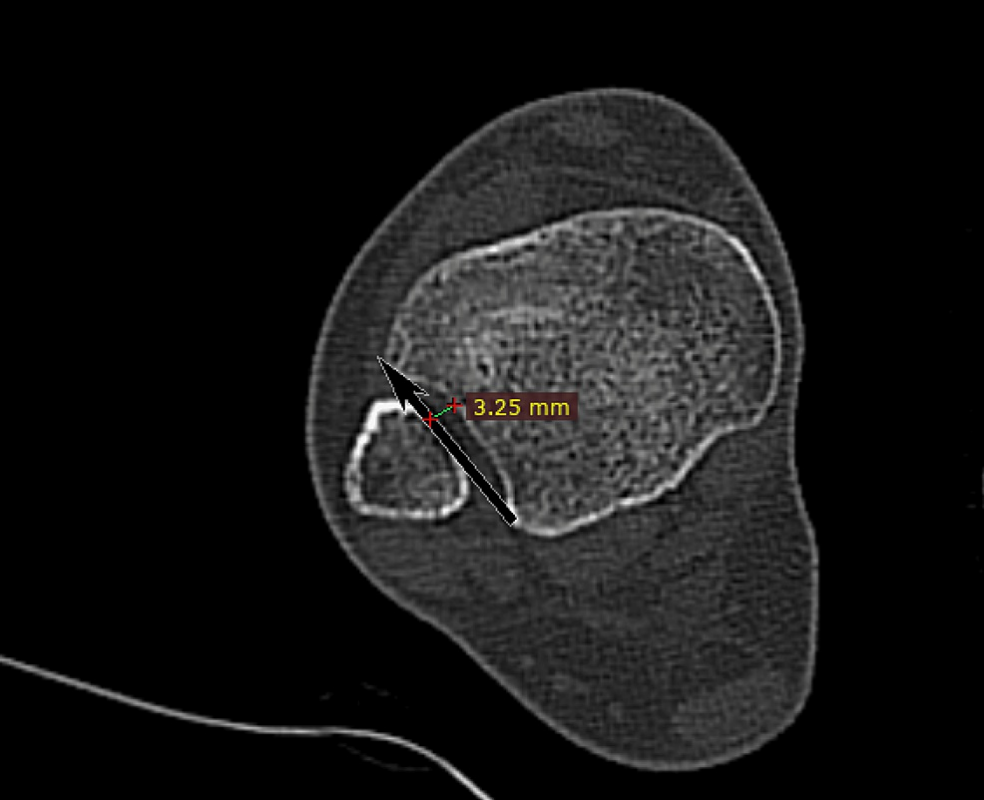
Incisura depth. Depth is the distance of the incisura line (black arrow) to the deepest point of the tibia groove.

Incisura depth: It is the distance from the deepest incisura point to the incisura line (Figure 2).

**Figure 3:**
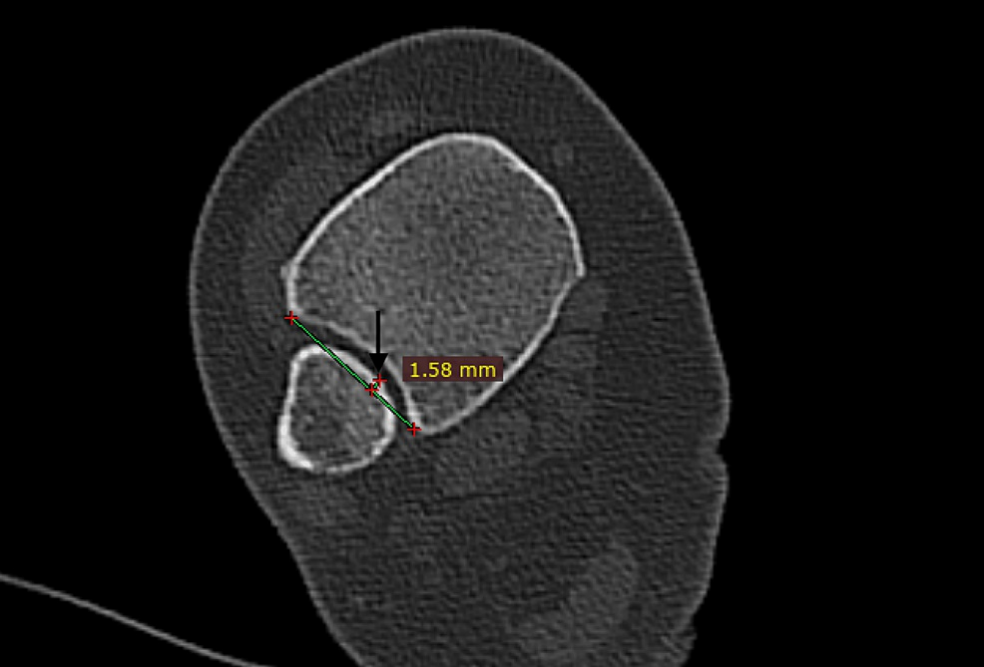
Fibula engagement. This is the distance (black arrow) of the most medial part of the fibula to the incisura line (green line). The value is positive if the fibula is engaged and negative if the fibula is outside the incisura line.

Fibular engagement is the distance of the most medial part of the fibula from the incisura line. Engagement has a positive value if the fibula is engaged in the incisura, and a negative one if the fibula is not engaged at all (Figure 3).

**Figure 4:**
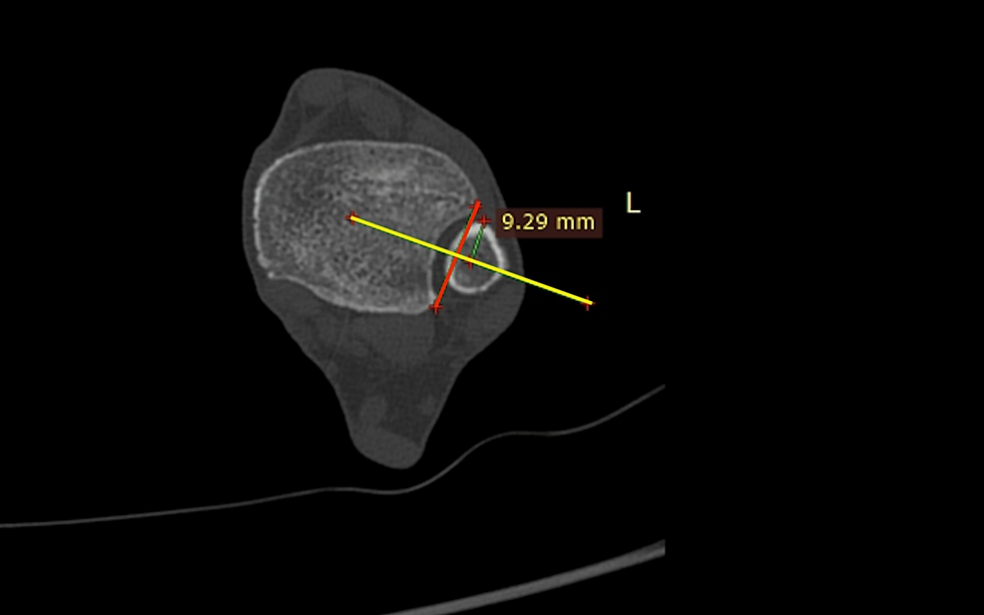
Fibula anterior translation. The distance of the most anterior point of the fibula from the baseline (yellow line).

Fibula anterior translation: The anterior translation was the distance from the most anterior fibula point to the baseline (Figure 4).

Measurements of the ankle joint

**Figure 5:**
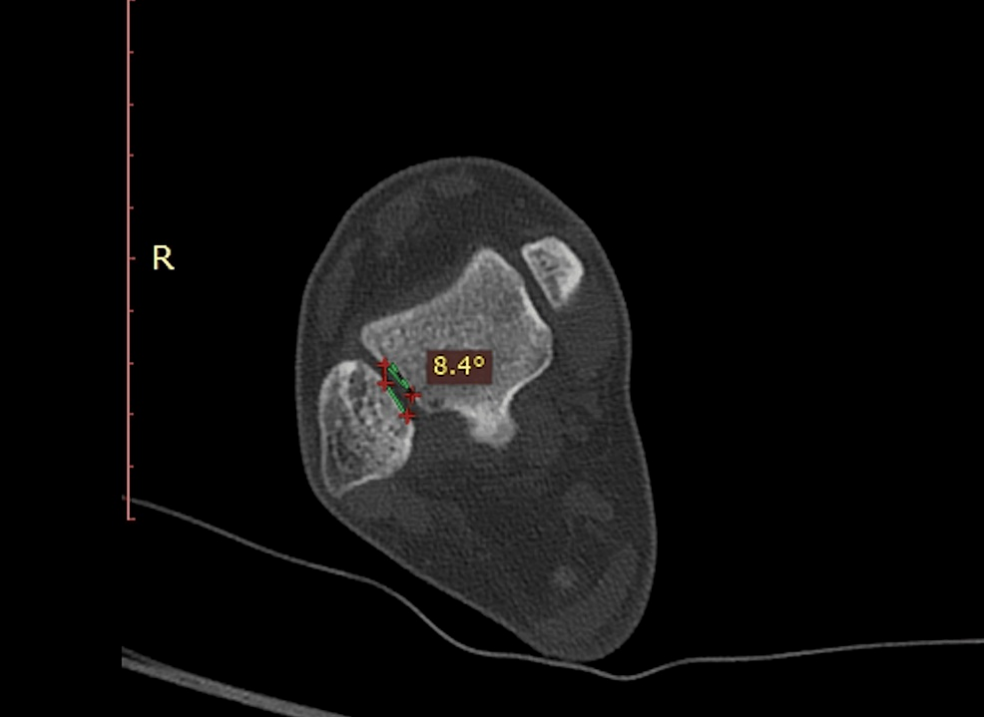
Torsion. The Cobb angle between the lateral malleolus and lateral talar facet. The measurement has a positive value in the anteverted fibula and a negative value in the retroverted fibula.

Fibula torsion: It assesses the rotational relationship between the lateral malleolus and lateral talar facets. The evaluation was made at a lower level, where both facets were visible. The angle in between was the fibula torsion (Figure 5).

**Figure 6:**
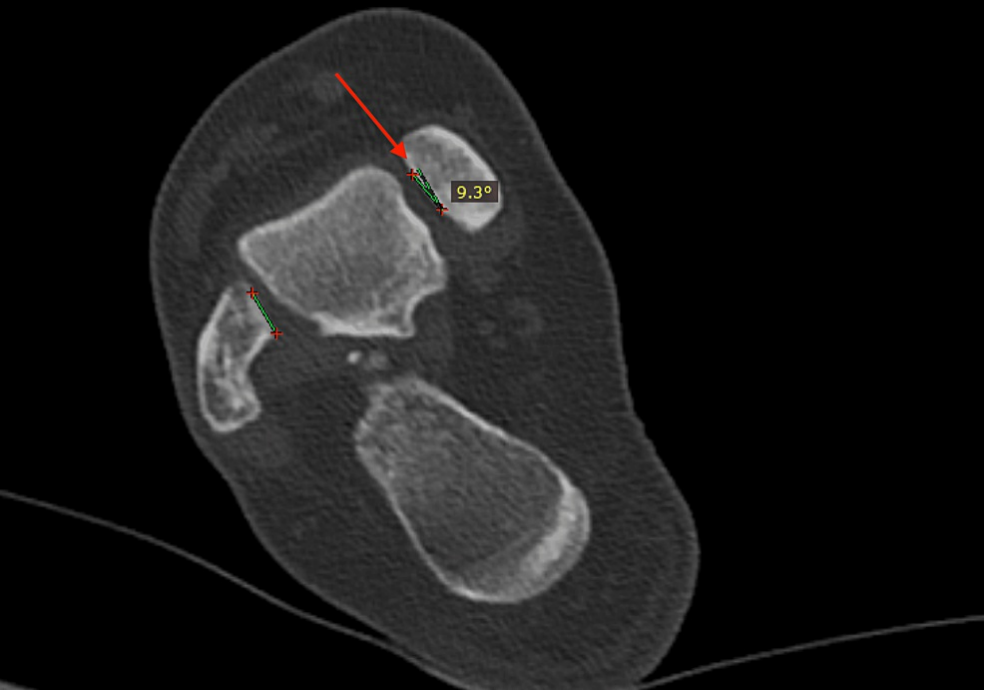
Relative torsion. The Cobb angle (red arrow) between the lateral and medial malleolus facets. The measurement has a positive value if the fibula is externally rotated against the medial malleolus and a negative value if it is internally rotated.

Relative torsion: This measurement investigated the rotational relationship between the lateral and medial malleolus facets. The assessment was at the level where both facets were visible. The angle in between was the relative torsion (Figure 6).

All examinations were made on the RadiAnt DICOM viewer (version 1.9.16, 32-bit; Medixant Company, Poznan, Poland) using the 3D multiplanar reconstruction (MPR) protocol [17]. The measurements were executed on proper axes and were evaluated by two independent investigators. The statistical analysis was made on Statistical Product and Service Solutions (SPSS, version 23; IBM SPSS Statistics for Windows, Armonk, NY). The level of significance was p<0.05.

## Results

Sixty normal ankles were enrolled in our study. Thirty-three of them (55%) were right, and 27 (45%) were left. The mean age of the subjects (36 women (60%) and 24 men (40%)) was 48.3 years old (SD=15.3 years). Interobserver Cronbach’s alpha reliability was 0.94, 0.94, 0.96, 0.97, 0.91, and 0.93 for the depth, incisura rotation, engagement, anterior translation, torsion, and relative torsion, respectively. 

The mean notch depth at the level of the syndesmosis is 3.92 mm (SD=0.99 mm), and the mean incisura rotation is 4.2 (SD=4.17°) of retroversion. Fibula anterior translation was measured at 8.87 mm (SD=1.7 mm), while tibiofibular engagement was calculated at 0.11 mm (SD=1.57 mm). The torsion between the fibula, and the talus was -3.05° (SD=4.17°), while the relative torsion was 6.2° (SD=6.06°). Accumulative data are shown in Table 1. 

**Table 1:** Measurement results. SD = Standard deviation.

Measurements	Mean value	SD	Min value	Max value
Notch depth (mm)	3.92	0.99	1.82	6.85
Notch rotation (deg)	4.2	4.17	-11.8	9
Fibula anterior translation (mm)	8.87	1.7	5	12.9
Tibiofibular engagement (mm)	0.11	1.57	-3.25	3.28
Torsion (deg)	-3.05	4.17	-12.7	8.3
Relative torsion (deg)	6.2	6.06	-12	16.9

**Figure 7:**
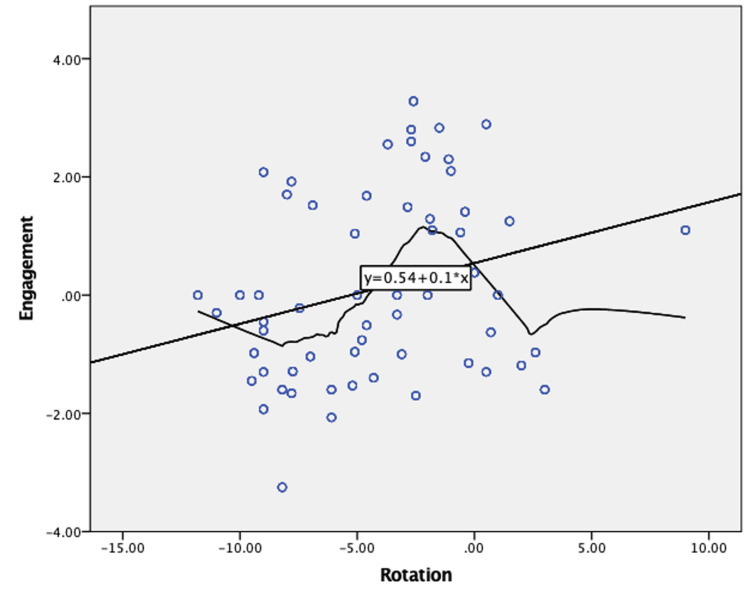
Correlation between incisura rotation and fibula engagement. Higher notch retroversion is related to greater fibula engagement.

After that, we investigated whether those physical measurements could correlate in between. The incisura retroversion positively correlated with the fibula engagement (Pearson correlation=0.273, p=0.03), meaning that patients with greater incisura retroversion had more engaged fibulas in the tibial groove (Figure 7).

Nevertheless, contrary to what was expected, this inherent incisura version did not affect the fibula anterior translation (Pearson correlation=0.13, p=0.1). Additionally, the incisura depth was not found to affect either the fibula anterior translation (Pearson correlation=0.019, p=0.08) or the fibular engagement (Pearson correlation=0.223, p=0.08). Notably, torsion and relative torsion had no correlation (Pearson correlation=0.12, p=0.3) (Table 2).

**Table 2:** Correlations of the measured parameters.

Correlations	Pearson correlation	P value
Notch rotation/fibula engagement	0.273	0.03
Notch rotation/fibula anterior translation	0.13	0.1
Notch depth/fibula anterior translation	0.019	0.08
Notch depth/fibula engagement	0.223	0.08
Torsion/relative torsion	0.12	0.3

## Discussion

In this study, we described the average radiological anatomy and anatomical relationships of the DTFJ. Our findings show that at 1 cm above the tibial plafond, the fibular notch is retroverted and the tibiofibular engagement is borderline. Additionally, the fibula articular facet is internally rotated against the talus but externally rotated compared to the medial malleolus facet at the ankle joint.

The DTFJ shape has been long studied, with a broad spectrum of anatomical variations being reported in the literature regarding its depth [18]. Although we did not characterize the notch as deep or shallow, we also found this diversity in our study, with our cohort’s notch depth ranging from 1.82 mm to 6.85 mm. Our calculated mean depth was 3.92 mm and is comparable with the findings of a cadaveric CT study by Ebraheim et al. [19], where the mean notch depth was measured at 4.29+/-1.26 mm.

Fibula anterior translation was 8.87 mm (SD=1.7mm, SE=0.22), measured by the same method as reported by Boszczyk et al. [15]. Although the authors of this study found a 27.8% rate of sagittal plane malreduction, they did not report their results in normal ankles. Notably, despite this measurement being adequate for anteroposterior translation evaluation, it cannot be clinically valuable as there is no way it can be intraoperatively calculated. Conversely, the distance of the most anterior fibula part to the most anterior tibial prominence can be clinically measured. Nevertheless, Cherney et al. [20] used this measurement for comparison between normal and operated ankles, unfortunately not reporting the distribution of this parameter in normal ankles. Clinically, this fibula translation malreduction is quite common and influenced by the reduction technique, especially when clamps are used [21,22]. Again, we strongly believe that the surgical exploration of the anterior notch corner and restoration of the anterior tibial prominence and fibula relationship during syndesmotic fixation resolves this issue. 

Under the same concept, coronal malreduction has also been debated. We found that the incisura depth and fibula engagement do not correlate in normal ankles (p>0.05). Conversely, Rushing et al. proved that coronal malreduction is a real phenomenon by means of over or under-tightening of the DTFJ [23]. At 1 cm above the tibial plafond, the tibiofibular joint engagement is borderline at +0.11 mm with an SD of 1.57 mm. Those data refer to the mean values, meaning that the engagement could be either positive or negative, and this cannot be predicted for each patient separately. Thus, as depth and engagement are irrelevant, we agree with Haynes et al. [24] that the reduction should not be forced to overcompression as it may result in high malreduction rates.

We found no correlation between the notch rotation (version) and the anterior translation of the fibula. These results contradict Boszczyk et al.’s findings [15]. The authors of this study compared the incisura version of the healthy ankles to the position of the contralateral operated fibula, finding that patients with incisura anteversion can experience excessive fibula anterior translation and vice versa. While essential, this does not apply to the true anatomical relationship between the tibia and fibula in non-injured ankles. 

In our analysis, fibula torsion was measured at -3.05°, which means that the lateral malleolus facet is internally rotated to the lateral talar facet with the foot in a plantigrade position. In the DTFJ, the fibula translates to cephalad and externally rotates as the foot moves from plantarflexion to dorsiflexion [25]. Thus, foot positioning is of high importance when trying to rotationally fix the fibula. In our former study, we tried to place the lateral malleolus facet parallel to the lateral talar facet, resulting in excessive external rotation [14]. Our recent findings suggest that the fibula should be slightly internally rotated against the talus with the foot in a plantigrade position. In most cases, the DTFJ is stabilized with either syndesmotic screws or other elastic fixation means [26]. This rotational malreduction is additionally influenced by the direction of the syndesmotic screws and the inherent fibula anatomical shape [27]. Nevertheless, those changes seem to be temporary and resolved after the screw removal [28].

Finally, we found that torsion and relative torsion did not correlate (Pearson correlation=0.12, p=0.3). This finding is chiefly explained by the anatomical talar shape and the angulation between the lateral and medial talar facets [29] and the fact that the medial malleolus is a fixed structure compared to the mobile fibula. Thus, it seems that we cannot force change in relative torsion when fixing bimalleolar fractures or we compromise the talar tilt because of the iatrogenic mortise incongruency [30]. Additionally, anatomical fixation of the medial malleolus is critical as malreduction leads to a 27.8% decrease in the tibiotalar contact area [22]. For this reason, we always fix the medial malleolus first and then proceed to the fibula and DTFJ reduction, as the lateral structures are tolerant to rotational amendments.

Our study has provided significant data about the normal radiological anatomy of the DTFJ. In ankle fracture fixation, surgeons should aim to restore the normal anatomy, and our findings contribute to this direction. Nevertheless, there are several limitations. First, we evaluated the normal DTFJ anatomy 1 cm above the tibial plafond. In clinical practice, ankle fractures can involve the DTFJ at a lower level than that. Additionally, different limb lengths and patient heights could be significant limiting factors compromising the homogeneity of our results. Second, all measurements were made with the foot in the plantigrade position. The DTFJ, though, is a mobile structure. This means we cannot know how all measurements change and whether the proven correlations are verified throughout the physiological motion of the ankle joint. Finally, our results provide the mean and border values of our measurements. This does not mean that trying to achieve a reduction according to our results can provide exact and reproducible outcomes for each patient separately. The wide anatomical variations of the DTFJ pose a significant limitation in clinical practice.

## Conclusions

At 1 cm above the tibial plafond line, the notch is expected to be retroverted, the fibula anteriorly translated, and borderline engaged. For the ankle joint, the fibula is internally rotated against the talus but externally rotated relatively to the medial malleolus facet. To our knowledge, this is the first study that describes the normal radiological anatomy of the DTFJ in the normal population. The results of our study contribute to clinical practice as a guide for surgeons. We believe that trying to reduce and restore the tibiofibular relationship according to our data can provide high-reduction quality and better clinical outcomes.
